# Digital animation as a tool to enhance informed consent when recruiting infants with biliary atresia to a clinical trial

**DOI:** 10.1002/jpn3.70190

**Published:** 2025-08-12

**Authors:** Sara Mancell, Fiona Lavelle, Salma Ayis, Anil Dhawan, Kevin Whelan

**Affiliations:** ^1^ Department of Nutritional Sciences King's College London London UK; ^2^ Department of Nutrition & Dietetics King's College Hospital NHS Foundation Trust London UK; ^3^ Department of Population Health King's College London London UK; ^4^ Paediatric Liver, GI and Nutrition King's College Hospital NHS Foundation Trust London UK

**Keywords:** information tools, recruitment, research ethics, video

## Abstract

**Objectives:**

Participants may have a poor understanding of the research they are involved in due to the challenges of receiving information during acute illness and the complexity and length of participant information sheets. This study aimed to assess the impact and acceptability of using digital animation to recruit infants with biliary atresia to a clinical trial.

**Methods:**

The mixed method design used questionnaires and interviews to assess the feasibility of using animation during recruitment to a feasibility trial (ISRCTN81936667). All participants received verbal and written information, and after the animation was introduced, participants additionally received the animation. Quantitative data are presented descriptively (median, frequencies), and recruitment before and after introducing the animation was compared using Fisher's exact test. Qualitative data were analysed thematically, and the combined quantitative and qualitative results were considered together.

**Results:**

Perceptions of the animation were highly positive, with between 81.3% to 100% agreeing with positively framed statements and 87.5% to 100% disagreeing with negatively framed statements. However, there was no difference in numbers consenting to participate before (14/16, 87.5%) and after (16/18, 88.9%) introducing the animation (*p* = 1.00). Three qualitative themes emerged relating to the animation: technical accessibility, cognitive accessibility and enabling understanding.

**Conclusions:**

The animation was viewed positively by participants who felt it increased understanding and enabled them to share information with others. Although this improved the informed consent process, it did not impact the consent rate. Digital animation could represent an effective way to present study information, better enabling participants to provide valid informed consent.

**Clinical Trial Registration:**

Trial identifier: ISRCTN81936667

## Abstract (highlights)

**What is Known:**

- Participants may have a poor understanding of the research they participate in due to the challenges of receiving information during illness and the complexity and length of participant information sheets.
- Digital animation may help to improve informed consent to clinical trials.

**What is New:**

- Animation helped increase understanding of study information and enabled the sharing of this information with others.
- Animation may help those with comprehension difficulties and non‐English speakers understand study information.
- The stress experienced by parents of infants with biliary atresia after diagnosis may impact their capacity to access and cognitively process study information.

## 1 INTRODUCTION

Undertaking research in infants with a new diagnosis of a serious illness, such as biliary atresia, is important to ensure evidence is available for optimal management during this critical period. This often requires recruiting infants at a time of profound stress for parents. There is a legal and ethical requirement to ensure participants have the information needed to provide informed consent,^1^ defined as consent given voluntarily by a competent person adequately informed about what it means for them (or their child) to participate.^2^ Despite this, participants have been found to have a poor understanding of important aspects of research.^3^ In addition to the ethical and legal issues this presents, *un*informed consent could impact adherence and retention as participants may not understand what is required of them.^4^

The complexity and volume of participant information sheets have been identified as barriers to understanding.^5^, ^6^ Participant information sheets have become longer over time^7^ due to legal aspects of trials^5^ with one review finding that the average participant information sheet was 19 pages.^8^ Longer documents are associated with reduced understanding^9^ partly due to incomplete reading,^10^ and there is a risk this could affect recruitment as decisions not to participate may be based on incomplete information.^11^ It is essential that recruitment is optimised; meanwhile, there is an ethical and legal obligation that information is accessible to ensure comprehension of what participation entails.

In view of the challenges of long, paper‐based, participant information sheets, digital media (including animation) has been used to improve informed consent.^12^, ^13^, ^14^, ^15^ Animation may better meet the needs of participants who are increasingly accustomed to digital information,^12^ and digital media is associated with increased satisfaction and understanding of trial information^16^; however, most studies are in adults. To our knowledge, no studies have reported using animation to improve parent understanding as part of recruitment to trials involving infants with gastroenterological or hepatological disease.

Biliary atresia is a rare liver disease occurring in 1 in 15,000–20,000 live births^17^ making it challenging to recruit sufficient infants to trials.^18^ An additional challenge is that recruitment early after diagnosis occurs at a highly stressful period for parents.^19^ It is therefore essential to ensure parents have all the information required in an accessible format to enable them to provide valid informed consent to participate in research.

The aim was to investigate the impact and acceptability of using animation to recruit infants with biliary atresia to a feasibility randomised controlled trial (RCT).

## 2 METHODS

### 2.1 Ethics statement

This study was approved by the London‐Dulwich Research Ethics Committee (22/LO/0822) and the Health Research Authority and Health and Care Research Wales and was registered with ISRCTN before commencing recruitment (ISRCTN81936667).

### 2.2 Participants and study design

This study included all infants recruited to a feasibility RCT between January 2023 and March 2025 who had undergone Kasai portoenterostomy surgery for biliary atresia at King's College Hospital NHS Foundation Trust at or before 12 weeks of age.^20^ The study being conducted was a feasibility RCT of medium‐chain triglyceride supplementation.^20^ Initially, parents were provided with verbal and written information in the form of a participant information sheet (information sheet group), however, during the recruitment period an animation was developed and provided additionally (animation group). For the purposes of this study, ‘participants’ refers to parents and not the infants participating in the study.

### 2.3 Developing the animation

The animation was co‐designed with stakeholders, including health professionals and a public and patient involvement panel comprised of parents of children with biliary atresia. The animation was developed using Vyond software 2024 (Go Animate Inc.) and refined by these stakeholders. The final version was 2 min and 20 s and described what would be expected of participants and aspects such as randomisation, risks and benefits. Parents were invited to access the animation using a quick response (QR) code or through a website link https://youtu.be/u0ysqYblY-E (Figure S1).

### 2.4 Design of the evaluation of the animation

This mixed methods sequential explanatory study^21^ included recruitment and retention rates, quantitative questionnaires and qualitative interviews to assess the acceptability and impact of using animation to recruit infants with biliary atresia to the feasibility RCT.^20^ The qualitative component was used to help explain findings from the quantitative component and provide more in‐depth insights than would have been possible with questionnaires alone.^22^ The animation group completed a questionnaire after watching the animation and answered interview questions at the end of the study (after 5 weeks). Parents who declined participation were still invited to complete the animation questionnaire and be interviewed. Data collection was between January and December 2023 (information sheet group) and January 2024 and March 2025 (animation group).

### 2.5 Measures

No validated tool was identified to assess the animation's impact or acceptability; therefore, a questionnaire was developed with help from stakeholders (health professionals, public and patient involvement panel). The questionnaire consisted of 12 questions relating to the first three levels of the Kirkpatrick model of evaluation to cover satisfaction and engagement (reaction), knowledge (perceived learning) and behaviour based upon learning (behaviour).^23^, ^24^ Two additional questions related to the animation's acceptability. Responses were recorded using a five‐point Likert scale (strongly agree to strongly disagree), and a free‐text section was included for comments. The fourth level of the Kirkpatrick model, progress towards outcomes (results)^23^ related to recruitment and retention rates before and after introducing the animation. Participant views were explored further during semi‐structured interviews using a topic guide (Table S1).

### 2.6 Data analysis

Descriptive data are presented as median (interquartile range) for continuous variables and frequencies (%) for categorical data. Recruitment and retention rates were compared using Fisher's exact test using STATA version 17.0 (StataCorp LLC).

Interviews and the free‐text section of the questionnaire were transcribed verbatim and de‐identified to ensure confidentiality. During analysis, the researcher (SM, paediatric dietitian) considered their impact on results.^25^ Qualitative software f4transkript version 8.3 and f4analyse version 3.4.5 (GmbH) were used to assist with manual transcription and coding. Data familiarisation was achieved through reading and re‐reading, and two researchers (SM and FL, behavioural scientists) independently coded all transcripts. There was high agreement, with discrepancies discussed and agreement reached on all codes. The codes were grouped into themes (SM) and refined through critical discussion (SM, FL and KW) to ensure clear distinctions, and quotes demonstrated typical views within each theme. The sample was considered to have sufficient information power,^26^ given the focused aim and tight specificity (parents of infants with biliary atresia), and the experience of the interviewer generated focused and rich data. Using a synergistic approach,^27^ quantitative and qualitative findings were combined to identify common findings.

## 3 RESULTS

There were 30 infants (18 male and 12 female) recruited to the feasibility RCT, 14 (46.7%) before the animation (information sheet group) and 16 (53.3%) after the animation was introduced (animation group). Characteristics of included infants are shown in Table S2.

### 3.1 Questionnaire responses

All 16 parents completed the questionnaire (Table 1 **)**. The two parents who declined participation did not wish to complete the questionnaire (or be interviewed). Overall, responses were positive, with 81.3%–100% agreeing/strongly agreeing with positively framed statements and 87.5%–100% disagreeing/strongly disagreeing with negatively framed statements.

**Table 1:** Questionnaire responses in relation to the digital animation grouped by reaction, perceived learning and behaviour (*n* = 16).

Questionnaire items	Strongly agree, *n* (%)	Agree, *n* (%)	Neither agree nor disagree, *n* (%)	Disagree, *n* (%)	Strongly disagree, *n* (%)
Reaction					
The images used in the animation were appropriate	12 (75.0)	4 (25.0)	0 (0.0)	0 (0.0)	0 (0.0)
The quality of the images used in the animation was good	13 (81.3)	3 (18.8)	0 (0.0)	0 (0.0)	0 (0.0)
The amount of time it took me to watch the animation was acceptable	12 (75.0)	4 (25.0)	0 (0.0)	0 (0.0)	0 (0.0)
The animation was difficult to access (e.g., using the QR code, viewing on YouTube)	0 (0.0)	0 (0.0)	1 (6.3)	6 (37.5)	9 (56.3)
Perceived learning					
The animation was easy to understand	11 (68.8)	5 (31.3)	0 (0.0)	0 (0.0)	0 (0.0)
The animation helped me learn about the study	12 (75.0)	3 (18.8)	1 (6.3)	0 (0.0)	0 (0.0)
The amount of information in the animation was acceptable for me to understand	13 (81.3)	3 (18.8)	0 (0.0)	0 (0.0)	0 (0.0)
There was some information missing from the animation	0 (0.0)	0 (0.0)	2 (12.5)	7 (43.8)	7 (43.8)
Behaviour					
The animation helped me to decide whether to take part in the study	10 (62.5)	3 (18.8)	3 (18.8)	0 (0.0)	0 (0.0)
Watching the animation made me want to take part in the study	10 (62.5)	5 (31.3)	1 (6.3)	0 (0.0)	0 (0.0)
The animation helped me to explain the study to others (e.g., family or friends)	12 (75.0)	4 (25.0)	0 (0.0)	0 (0.0)	0 (0.0)
The animation helped me to think of questions about the study to ask the researcher	7 (43.8)	7 (43.8)	1 (6.3)	0 (0.0)	1 (6.3)
Overall acceptability					
Overall, I was pleased that I was offered this animation	11 (68.8)	5 (31.3)	0 (0.0)	0 (0.0)	0 (0.0)
I think this animation should be used to provide information about the study to other families	11 (68.8)	5 (31.3)	0 (0.0)	0 (0.0)	0 (0.0)

Abbreviation: QR, quick response.

In terms of reaction, participants agreed on the acceptability of images and the animation's duration (75% strongly agreed, 25% agreed). For perceived learning, participants agreed the animation contained the right amount of information (81.3% strongly agreed, 18.8% agreed) while for behaviour, they agreed it helped explain the study to others (75% strongly agreed, 25% agreed). In terms of acceptability, participants were pleased to have been offered the animation and agreed it should be used in the future (68.8% strongly agreed, 31.3% agreed).

Recruitment and retention rates were compared (Table 2). The consent rate was 87.5% (14/16) before introducing the animation and 88.9% (16/18) after (*p* = 1.00). All infants in the information sheet group commenced the study compared to 87.5% (14/16) in the animation group (*p* = 0.49), and study completion was 85.7% (12/14) in the information sheet group compared to 81.3% (13/16) in the animation group (*p* = 1.00).

**Table 2:** Recruitment and retention for the feasibility RCT for the information sheet and animation (*n* = 30).

Outcome	Information sheet (*n* = 14)	Animation (*n* = 16)	*p* *
Recruitment, *n* (%)			
Consented to take part in the study	14/16 (87.5)	16/18 (88.9)	1.00
Retention, *n* (%)			
Commenced the study	14/14 (100)	14/16 (87.5)	0.49
Completed the study	12/14 (85.7)	13/16 (81.3)	1.00

Abbreviation: RCT, randomised controlled trial.

* *p* Values are the result of Fisher's exact test.

### 3.2 Free‐text comments and interview data

Nine (56.3%) participants from the animation group were interviewed, and four (25.0%) made comments in the questionnaire's free‐text section. Three themes were identified: (i) technical accessibility, (ii) cognitive accessibility and (iii) enabling understanding.

1. Technical accessibility
  Participants described technical aspects that influenced the animation's accessibility. Access to the animation was thought to be straightforward, and participants described the ease of sharing it with family. A common view was that the animation was a suitable length (2 min, 20 s) and that this was important to ensure participants watched it fully.
  I think it was very good it was short because some people…wouldn't bother to…watch the whole thing if it was too long. That's what we're like, we're very impatient…I think it really, really did explain everything. (Participant 023)
  Satisfaction was high, with participants expressing that the animation was visually pleasing.
  There are…fantastic pictures. I really liked them…It's amazing!…I think as humans as soon as you…see cartoons and colours it's good. (Participant 027)
2. Cognitive accessibility
  Participants described cognitive aspects that influenced accessibility. The language was described as clear and without complicated words that could have limited understanding.
  …it was very clear. No difficult words. No medical words that would have thrown us off. Because we've been hearing all sorts. I mean with his medications some of them I can't even pronounce. So, things like that are really intimidating. (Participant 023)
  Some participants described preferring visual information and the challenge of comprehending verbal or written information. They felt the animation should be offered alongside other information sources to allow for individual preferences and learning needs.
  I showed it to my mum ‐ she understood it. She's not very good at reading paper… so she understood it much better…She's got dyslexia…to have both is great because some people read and understand and some people like to listen and understand. (Participant 030)
  Information overload was described in terms of other information that parents faced in a stressful period after diagnosis. A lack of time and a focus on information related to their baby's care were cited as reasons for not accessing the animation.
  We didn't see the video… We didn't have the courage to go through all this information… I didn't take my phone to scan the QR code… our mind and our focus was on [doctor's name] to tell us everything. (Participant 025)
3. Enabling understanding
  Participants discussed the ease of understanding the animation and how this influenced participation.
  It helped in understanding it more. Because verbally you can tell us, but we saw and the animation is almost like, oh ok, this is exactly what you mean. (Participant 023)

In contrast, some participants indicated they had already understood the same information provided verbally and in writing.

I thought it was the same information as what you'd given us on paper…I understood the paper so the animation I didn't need as much. (Participant 030)

Some participants felt the animation could increase understanding for those whose first language was not English.

Even my wife [non‐native speaker] understood it. I felt when I was telling her about it, she was like ‘I'm not sure, I'm not sure.’ But as soon as she saw the video it made so much sense. Just by watching the video she understood it… (Participant 027)

Participants varied in their views of whether watching the animation helped them decide they wanted to participate.

…when you first came, we didn't really understand it and then you gave us the booklet and we watched the video and it kind of made more sense and we thought ok yeah let's take part… (Participant 033)

I already wanted to take part…before I was shown the animation. (Participant 030)

### 3.3 Synthesis of quantitative and qualitative results

**Figure 1:**
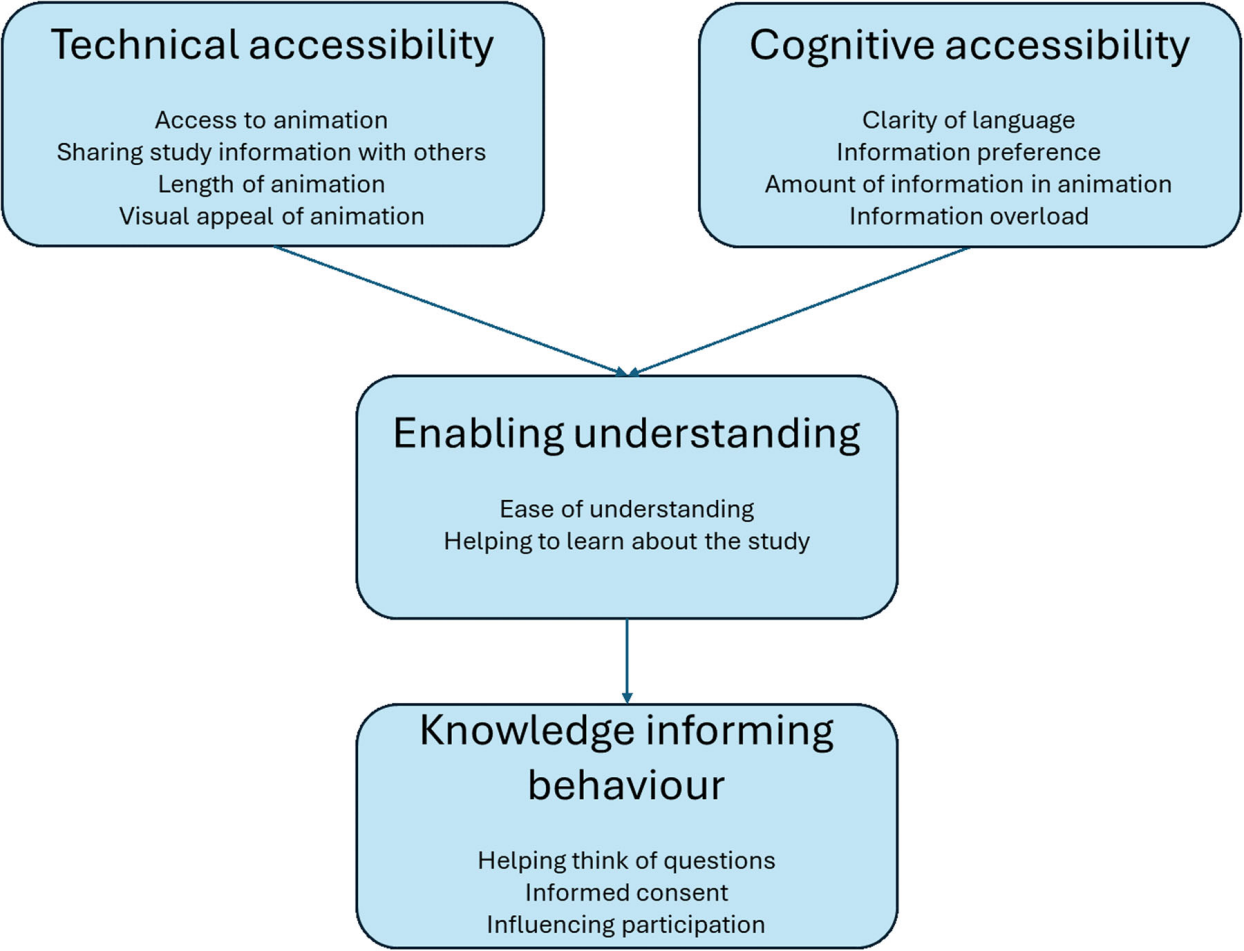
Synthesis of quantitative and qualitative findings related to the animation. The technical and cognitive accessibility of the animation were important elements that enabled understanding of the study. In some cases, the increase in understanding from the animation helped parents think of questions and decide whether to participate.

The combined quantitative and qualitative findings are shown in Figure 1. The accessibility of the animation was considered important and facilitated participants being able to watch and share the animation and feel satisfied with the content. The information provided was deemed acceptable; however, information overload was highlighted as a barrier. Participants felt the animation helped them understand the study thanks to the clear content, and in some cases, the animation helped parents think of questions and decide whether to participate.

## 4 DISCUSSION

Informed consent for inclusion in trials is essential, but participants and in particular parents of unwell infants, can feel overwhelmed when presented with information.^28^, ^29^ Our aim was to investigate the acceptability and impact of using a digital animation to recruit infants with biliary atresia to a feasibility RCT. We found that the animation was viewed positively by participants who felt it increased their understanding of the study and enabled them to share this information with others. However, it did not significantly impact recruitment or retention. The view was that the animation should be offered alongside verbal and written information to account for personal preferences.

The lack of impact of animation on recruitment and retention is similar to other studies.^12^, ^13^, ^14^, ^16^, ^30^, ^31^ A Cochrane review of 16 trials investigating audio‐visual interventions (one of which used animation) reported no impact on recruitment rate,^16^ although similarly to this study, audio‐visual interventions were associated with satisfaction and improved understanding, a central tenet of informed consent. In contrast, a meta‐analysis of three studies recruiting children to clinical trials found that recruitment increased by 54% when multimedia information (including animation) was used to present information versus when written information was used and no effect when both were provided together.^15^ However, the authors concluded that there was uncertainty about the true effect and more research is required, particularly involving children.^15^ The lack of an effect in the current study may relate to the relatively small sample size and therefore high risk of type II error, and ceiling effects due to high recruitment (88.2%) and retention rates (83.3%).

Participants in this study considered the animation easy to understand; in some cases, it was thought to help those with comprehension difficulties and those who did not speak English. The increase in understanding may be due to providing both verbal and visual information, which may help with information processing^32^ and memory development,^33^ while the non‐threatening aspects of animation^34^, ^35^ may be important for knowledge acquisition.^36^ Animation may also help to overcome barriers related to language and culture which may impact on understanding.^34^, ^37^

Key facilitators to enabling understanding were the animation's accessibility, including its short duration. In a study where parents viewed a parenting trial video, only 32% (*n* = 16) watched it in full, and on average, participants watched approximately 60% (3 out of 5 min) of the video.^31^ The likelihood of watching a video completely correlates with its length, with a predicted in‐video drop out of 53% for a 5‐min video and 71% for a 20‐min video.^38^ One study described both showing the animation to patients directly and providing a link to the animation.^39^ This could be relevant when recruiting infants with biliary atresia, where there are potentially high demands on parent time, meaning they may not access an animation independently or may be interrupted when viewing it. Watching, complete watching and re‐watching of the animation were not recorded in this study.

The cognitive accessibility of the animation was an important theme in this study, with information overload acting as a potential barrier to understanding. The findings indicate that participants' capacity to access the animation and cognitively process complex information in either format was sometimes curtailed by the intensity of their situation. Similar challenges have been reported in studies recruiting neonates and children on intensive care units^40^, ^41^, ^42^ and children diagnosed with life‐limiting conditions.^43^ Providing parents in such situations with an animation could help to enhance comprehension and ensure fully informed consent, a researcher's responsibility.

A strength of this study was the involvement of a public and patient involvement panel with lived experience as parents of children with biliary atresia. The mixed methods approach enabled participant views to be explored in depth, an essential step towards assessing recruitment preferences as part of feasibility testing for a future RCT. A limitation was that despite being invited to provide feedback, the views of parents who declined participation were not represented in this study. Another limitation was that the small sample size for the quantitative analysis made it difficult to draw comparisons and to generalise, although the qualitative analysis had information power. Finally, access to the animation was not randomised, although participants were not selected for the animation group, and all who could be provided with it were. Future studies could assess participant views on the inclusion of background music in animations, the narration style used and whether participants watched the animation in full. It would also be useful to consider parent characteristics, including education level, which could influence the understanding of study information.

## 5 CONCLUSION

To our knowledge, this is the first study to evaluate the impact and acceptability of using animation as part of recruitment of infants with biliary atresia. Participants reported highly positive experiences of the animation and thought it should be provided alongside verbal and written information; however, there was no impact on recruitment and retention. Mixed methods demonstrated beliefs that the animation would improve trial understanding, a central tenet of informed consent. Animation could, therefore, represent an effective and acceptable way to present information, enabling participants to fully understand a study and provide valid informed consent, which is an ethical and legal obligation for all researchers.

## CONFLICT OF INTEREST STATEMENT

The authors declare no conflicts of interest.

## Supporting information


**Figure S1. QR code and link for accessing animation.** Parents were invited to access the animation using the QR code or through the website link.

**Table S1. Topic guide.** The topic guide includes the questions used in semi‐structured interviews with parents in the animation group.

**Table S2. Characteristics of 30 infants included in the feasibility study.** This table includes all infants included in the feasibility study. Characteristics are shown for those in the information sheet group and animation group.
