# Supplementary material for: Digital animation as a tool to enhance informed consent when recruiting infants with biliary atresia to a clinical trial
Source: J Pediatr Gastroenterol Nutr. 2025 Aug 12;81(5):1242–50. doi: 10.1002/jpn3.70190 (PMC12580458; doi:10.1002/jpn3.70190)
Supplement: Supplementary file 1 — Figure S1. QR code and link for accessing animation. Parents were invited to access the animation using the QR code or through the website link. [file JPN3-81-1242-s001.docx]

**Supplemental Figure S1: QR code and link for accessing animation**

**Download using QR code:**

**
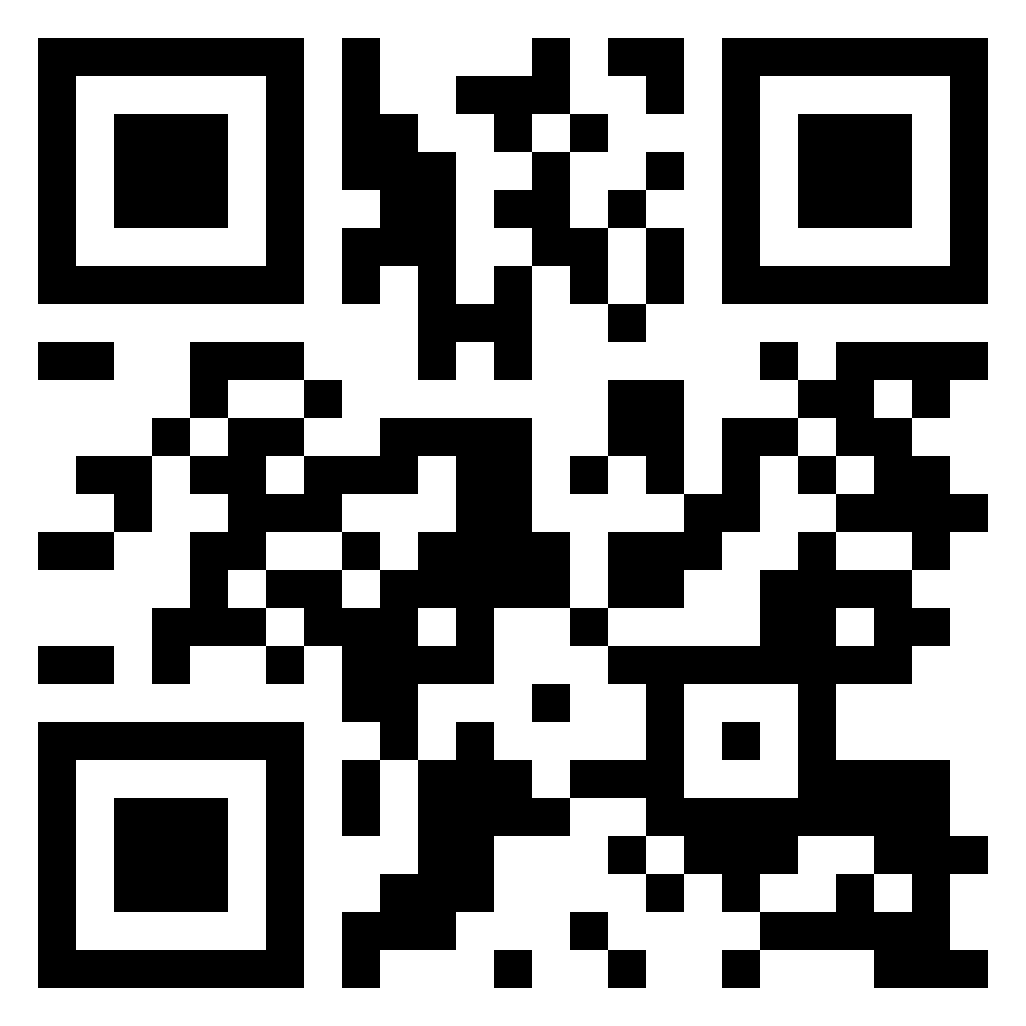
**

***or***

**Paste the link below into your browser:**

https://youtu.be/u0ysqYblY-E
