# Supplementary material for: Digital animation as a tool to enhance informed consent when recruiting infants with biliary atresia to a clinical trial
Source: J Pediatr Gastroenterol Nutr. 2025 Aug 12;81(5):1242–50. doi: 10.1002/jpn3.70190 (PMC12580458; doi:10.1002/jpn3.70190)
Supplement: Supplementary file 2 — Table S1. Topic guide. The topic guide includes the questions used in semi‐structured interviews with parents in the animation group. [file JPN3-81-1242-s002.docx]

**Supplemental Table S1: Topic guide**

| **Item** | **Question** |
| --- | --- |
| 1 | What did you think about the animation for the study? |
| 2 | Did the animation help you to understand what the study was about? |
| 3 | Was there any more information you think we should include in the animation? |
| 4 | Is there anything we should change about the animation? |
| 5 | Do you think animation should be used in a future study about this topic? |
