# Supplementary material for: Digital animation as a tool to enhance informed consent when recruiting infants with biliary atresia to a clinical trial
Source: J Pediatr Gastroenterol Nutr. 2025 Aug 12;81(5):1242–50. doi: 10.1002/jpn3.70190 (PMC12580458; doi:10.1002/jpn3.70190)
Supplement: Supplementary file 3 — Table S2. Characteristics of 30 infants included in the feasibility study. This table includes all infants included in the feasibility study. Characteristics are shown for those in the information sheet group and animation group. [file JPN3-81-1242-s003.docx]

**Supplemental Table S2: Characteristics of 30 infants included in the feasibility study**

| Characteristics | All infants (n=30) | Information sheet only (n=14) | Animation group (n=16) |
| --- | --- | --- | --- |
| Sex of infant, *n* (%) |  |  |  |
| Male | 18 (60.0) | 11 (78.6) | 7 (43.8) |
| Female | 12 (40.0) | 3 (21.4) | 9 (56.3) |
| Age of infant at Kasai portoenterostomy, weeks, median (IQR) | 7.9 (6.0, 9.5) | 8.8 (6.8, 10.4) | 7.4 (6.0, 8.8) |
| Surgical classification of biliary atresia, *n* (%) |  |  |  |
| Type I/II | 0 (0) | 0 (0) | 0 (0) |
| Type III | 30 (100.0) | 14 (100.0) | 16 (100.0) |
| Clinical phenotype of biliary atresia, *n* (%) |  |  |  |
| Isolated | 22 (73.3) | 10 (71.4) | 12 (75.0) |
| Biliary atresia splenic malformation | 3 (10.0) | 1 (7.1) | 2 (12.5) |
| Cystic | 3 (10.0) | 2 (14.3) | 1 (6.3) |
| Cytomegalovirus Immunoglobulin M positive | 2 (6.7) | 1 (7.1) | 1 (6.3) |
